# Hypertrophic Stenosis of Pylorus Successfully Treated by Gastric Lavage

**Published:** 1905-09-23

**Authors:** 


### HYPERTROPHIC STENOSIS OF PYLORUS SUCCESSFULLY TREATED BY GASTRIC LAVAGE.

A practical illustration of the value of Dr. G. F. Still's advice that careful feeding and gastric lavage should be tried in cases of hypertrophic stenosis of the pylorus is afforded by a case reported by Dr. A. J. Blaxland.[1] The patient, a boy aged four months, was, when he came under observation in February 1905, an emaciated infant weighing only 8 lbs. He had the characteristic vomiting of pyloric obstruction, and, in addition, there was visible peristalsis, and a palpable tumour could be appreciated in the region of the pylorus. The feeding at first consisted entirely of "humanised" milk, 3 ozs. being given every two hours, the amount later being increased to 4 ozs. For the first four days vomiting occurred daily. The stomach was then washed out and this was continued twice a day for several weeks with the result that there was no vomiting for eleven days and only occasional rejection of the stomach contents later. The partial substitution of peptonised for "humanised" milk proved of value when the vomiting returned for a time, and by the end of May it was found sufficient to wash out the stomach twice a week. Under this treatment the general condition of the child improved enormously, the weight of the infant being increased from 8 to 12½ lbs. At the same time the visible peristalsis disappeared and the pyloric tumour was no longer palpable.

[1] Lancet, Sept. 16, 1905.

